# High Resolution Mapping of Enhancer-Promoter Interactions

**DOI:** 10.1371/journal.pone.0122420

**Published:** 2015-05-13

**Authors:** Christopher Reeder, Michael Closser, Huay Mei Poh, Kuljeet Sandhu, Hynek Wichterle, David Gifford

**Affiliations:** 1 Computer Science and Artificial Intelligence Laboratory, Massachusetts Institute of Technology, Cambridge, Massachusetts, United States of America; 2 Departments of Pathology and Cell Biology, Neurology, and Neuroscience, Center for Motor Neuron Biology and Disease, Columbia Stem Cell Initiative, Columbia University Medical Center, New York, New York, United States of America; 3 Genome Institute of Singapore, Singapore; 4 Department of Biological Sciences, Indian Institute of Science Education and Research (IISER), Knowledge City, Mohali, India; University of Crete, GREECE

## Abstract

RNA Polymerase II ChIA-PET data has revealed enhancers that are active in a profiled cell type and the genes that the enhancers regulate through chromatin interactions. The most commonly used computational method for analyzing ChIA-PET data, the ChIA-PET Tool, discovers interaction anchors at a spatial resolution that is insufficient to accurately identify individual enhancers. We introduce *Germ*, a computational method that estimates the likelihood that any two narrowly defined genomic locations are jointly occupied by RNA Polymerase II. *Germ* takes a blind deconvolution approach to simultaneously estimate the likelihood of RNA Polymerase II occupation as well as a model of the arrangement of read alignments relative to locations occupied by RNA Polymerase II. Both types of information are utilized to estimate the likelihood that RNA Polymerase II jointly occupies any two genomic locations. We apply *Germ* to RNA Polymerase II ChIA-PET data from embryonic stem cells to identify the genomic locations that are jointly occupied along with transcription start sites. We show that these genomic locations align more closely with features of active enhancers measured by ChIP-Seq than the locations identified using the ChIA-PET Tool. We also apply *Germ* to RNA Polymerase II ChIA-PET data from motor neuron progenitors. Based on the *Germ* results, we observe that a combination of cell type specific and cell type independent regulatory interactions are utilized by cells to regulate gene expression.

## Introduction

Regulatory regions that are scattered throughout the genome control the differential expression of genes in different cell types. One of the most well characterized types of regulatory regions is the enhancer [1]. Transcription factors bind to sequence motifs contained within an enhancer leading to increased transcription of one or more associated genes [2]. Several measurable characteristics of enhancers have led to the identification of hundreds of thousands of putative enhancers in the mouse genome [3]. Active enhancers have been shown to exhibit H3K27 acetylation [4, 5] and are often bound by the acetyltransferase p300 [6]. Chromatin at enhancers tends to be open [7] as reflected by DNaseI hypersensitivity. This corresponds to the ability of transcription factors to bind to enhancers. Mediator and cohesin have been shown to frequently bind enhancers [8] and are hypothesized to help stabilize chromatin loops that form to allow enhancers to interact with the genes that they regulate.

A single gene may be regulated by multiple enhancers in the same cell type, and such regulatory relationships have been shown to span large genomic distances [9]. Methods that predict active enhancers [10–16] have observed widespread changes in enhancer activity in different cell types [17]. It has been suggested that differential enhancer usage implements both cell-state specific and cell-state independent gene regulation [18].

To identify active enhancers and assign them to the genes that they regulate, we analyzed ChIA-PET [19] data for RNA Polymerase II (PolII). The chromatin interaction analysis by paired-end tag sequencing or ChIA-PET method combines chromatin immunoprecipitation to enrich for genomic locations occupied by a protein with chromatin conformation capture techniques to identify pairs of genomic locations that are spatially proximal in the nucleus. The resulting data provide information about chromatin interactions that involve a particular protein of interest. For the purpose of discovering high confidence chromatin interactions at high resolution from PolII ChIA-PET data we introduce *Germ*. This method utilizes a blind deconvolution step to model the positional noise in read pair alignments relative to locations of protein occupancy directly from the data. Another benefit of the blind deconvolution step is that a detailed model of the distribution of PolII occupancy is obtained simultaneously with the model of positional noise. *Germ* utilizes both models obtained through blind deconvolution to inform a model of joint protein occupancy which reflects the likelihood that any two genomic locations are simultaneously occupied by a single PolII instance. Such joint occupancy events reflect underlying chromatin interactions that involve PolII.

The most common approach to analyzing ChIA-PET data is implemented by the ChIA-PET Tool [20]. This approach discovers locations bound by a protein and interactions involving a protein through two separate, independent pipelines. In contrast to the approach taken by *Germ*, information about the occupancy of the protein is not used to refine the locations and sizes of the regions identified to be involved in chromatin interactions. Also, the ChIA-PET tool does not explicitly model the positional noise of read pair alignments relative to locations of protein occupancy other than by extending aligned locations by a heuristically determined number of base pairs.

We previously developed a method for analyzing ChIA-PET data called *Sprout* [21]. *Sprout* assumes that proteins occupy point locations and that ChIA-PET data reflect interactions only between such point locations. This assumption works well for factors such as CTCF that bind to the genome in a punctate fashion. PolII, however, is observed to occupy regions of variable width which are not accurately modeled by point locations. The assumption made by *Sprout* allows statistical power to be gained when modeling punctate binding factors while causing information to be lost when modeling PolII data. *Germ* preserves more detailed models of protein occupancy resulting in less loss of information. A benefit of this approach is that the density of protein occupancy can be queried for any location, not just the set of point locations that *Sprout* would identify as occupied.

We examined ChIP-Seq data for several enhancer-related factors to demonstrate that locations that are distal to annotated transcription start sites (TSSs) and are determined by *Germ* to interact with TSSs exhibit stronger enrichment for properties of active enhancers than corresponding locations discovered by the ChIA-PET Tool. Furthermore, the distal locations discovered by *Germ* to interact with TSSs align with locations enriched for active enhancer properties with very high spatial resolution. These findings support the analysis of PolII ChIA-PET data with *Germ* as a useful approach for identifying the locations of active enhancers at high resolution as well as pairing the identified enhancers with their regulatory targets.

By measuring transcription levels using RNA-Seq, we show that the number of enhancers that a gene interacts with is correlated with greater levels of transcription. We provide evidence that genes switch the enhancers that they interact with and that enhancers that are actively utilized in both cell types may in some cases switch the genes that they regulate. Finally, we compare the enhancers used by genes in embryonic stem cells (ESCs) and motor neuron progenitors (pMNs) and observe that cell type specific enhancers are enriched for cell type appropriate transcription factor motifs.

## Methods

### *Germ* Description

*Germ* is a novel method for analyzing ChIA-PET data that presents a detailed view of the occupancy of the genome by a protein of interest. *Germ* accomplishes this by modeling the distribution of self-ligation read pairs as a convolution of a model of the fragmentation process and an estimate of the marginal distribution of protein occupancy. The estimated marginal distribution is then used to inform the estimation of the joint distribution of protein occupancy. The estimated joint distribution reflects a detailed view of the likelihood that pairs of genomic locations are simultaneously occupied by a protein of interest.

**Fig 1:**
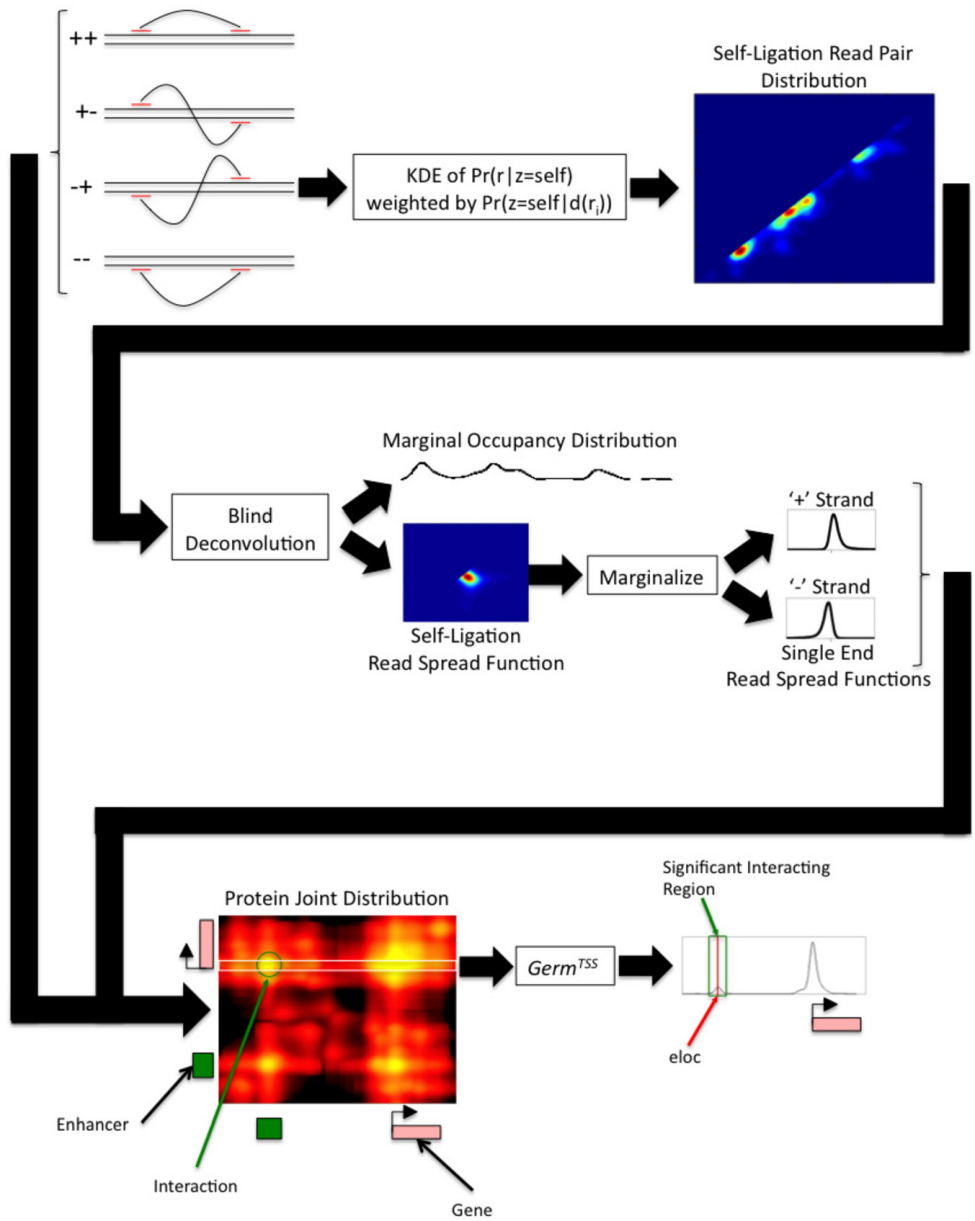
The workflow of *Germ* and *Germ* ^*X*^ Read pairs are aligned to the reference genome and read pairs are classified as ++, +-, -+, or—based on the strand to which the lower and higher coordinate ends of each pair align. A kernel density estimate of the self-ligation read pair distribution is constructed by weighting each -+ read pair by the estimated likelihood that it was produced by self-ligation. The marginal distribution of protein occupancy and the read spread function are recovered from the self-ligation read pair distribution through blind deconvolution. The estimated read spread function is marginalized in order to recover estimated single end read spread functions for each strand. The marginal distribution of protein occupancy, single end read spread functions, and inter-ligation read pairs are all used to estimate the joint distribution of protein occupancy. *Germ* ^*X*^ estimates the conditional distribution of protein occupancy for a set of genomic locations *X*. In the example shown, *X* is a set of annotated transcription start sites. A hypothesis test that is corrected for undersampling is applied to discover significant regions that are jointly occupied with a location in *X*. A location *eloc* within each interacting region is estimated to be the most likely jointly occupied location within the region.

*Germ* first estimates a two dimensional distribution over genomic coordinates that models the alignment of self-ligation read pairs (Fig 1). *Germ* explicitly models the effects of fragmentation in order to recover the marginal distribution of protein occupancy directly from the estimated self-ligation read pair distribution. *Germ* then uses the fragmentation model along with the marginal distribution of protein occupancy to estimate the two dimensional joint distribution of protein occupancy from the inter-ligation read pair alignments. *Germ* applies a hypothesis test for evaluating the significance of regions of the joint protein occupancy distribution to identify pairs of genomic regions that are likely to be jointly occupied by the protein over background levels of joint occupation.

We introduce a variation on *Germ* denoted *Germ* ^*X*^ for more efficiently identifying genomic regions that are jointly occupied by the protein with some location in a set of genomic locations *X*. A practical example of *Germ* ^*X*^ is to let *X* be a set of annotated transcription start sites in order to discover interactions between TSSs and enhancers by applying *Germ* ^*TSS*^ to RNA PolII ChIA-PET data. Finally, we describe a method that *Germ* ^*X*^ uses to estimate the amount of mass that is missing from the estimated joint distribution of protein occupancy because of undersampling of the distribution due to sequencing limitations. This allows the significance of interactions called by *Germ* ^*X*^ to be evaluated more accurately. We have included a table of notation (Table 1) to aid in our explanation of the *Germ* methodology.

**Table 1:** Notation.

Term	Definition
$r_{i}=\langle r_{i}^{\left(1\right)},r_{i}^{\left(2\right)}\rangle$	The aligned locations of the *i*th read pair
*R*	The set of all aligned read pair locations
*R*_*self*_, *R*_*inter*_	The sets of aligned self-ligation or inter-ligation read pairs
*z*_*i*_	The indicator of whether the *i*th read pair was produced by self-ligation or inter-ligation
*d*(*r*_*i*_)	The distance between the aligned locations of the *i*th read pair
*N*	The total number of aligned read pairs
*N*_++_, *N*_+−_, *N*_−+_, *N*_−−_	The number of aligned read pairs with a particular strand orientation
*N*_*self*_, *N*_*inter*_	The number of aligned self-ligation or inter-ligation read pairs
*K*_1_, *K*_2_	The standard univariate or bivariate Gaussian kernel
*h*_−+_, *h*_*non*−+_, *h*_*self*_	The bandwidth parameters for kernel density estimates
$ISE(\hat{f})$	The integrated square error of $\hat{f}$ relative to *f*
*q*_*i*_	The location occupied by the protein associated with the *i*th read pair
*RSF*(⟨*x* − *u*, *y* − *u*⟩)	The read spread function describing the probability of observing a self-ligation read pair *r* = ⟨*x*, *y*⟩ given *q* = *u*
⟨−*λ*, *λ*⟩	The peak of the estimated *RSF*
*reg*	A genomic region
*w*	The size (in base pairs) of *reg*
*p*	The probability of protein occupancy in *reg*
*Z*	A random variable representing the number of read pairs associated with *reg* according to the estimated distribution of occupancy
*Y*	A random variable representing the number of read pairs associated with *reg* according to the null model
*M*	The size of the mappable genome
*t*_*i*_	$=\sum_{u}\hat{\text{Pr}}(q=\langle u,v_{i}\rangle |R_{inter})$
*m*_*i*_	$=\hat{\text{Pr}}(q=v_{i})$
*τ*_*i*_	The estimated mass missing from *t*_*i*_
*f*	A significance threshold
*i*_*max*_	The index of the element in *X* with the greatest estimate mass
*c*	(*c*−1)*t*_*i*_*max*__ is an estimate of the total amount of mass that should be associated with *v*_*i*_*max*__
*eloc*	The location within a region that is jointly occupied with another region that has the greatest probability of being jointly occupied

#### Estimating the 2D Self-Ligation Read Pair Distribution

We assume that ChIA-PET linker tags have been removed from the read pair sequences, that read pairs that are known to have resulted from chimeric ligation events because they contain two different linker tags have been removed, and that the remaining linkerless read pairs have been aligned to the reference genome. Let *R* be the set of all aligned read pairs such that each read pair *r*_*i*_ ∈ *R* is represented by the pair of genomic coordinates to which the ends of the read pair align. We assume that the coordinates for each read pair are ordered so that if $r_{i}=\langle r_{i}^{\left(1\right)},r_{i}^{\left(2\right)}\rangle$, then $r_{i}^{\left(1\right)}\leq r_{i}^{\left(2\right)}$. We also assume that each read pair has an associated label according to the chromosome strands to which the ends align. There are four possible strandedness labels given the imposed ordering on the read pair ends. They are ++, -+, +-, and –. As mentioned above, all self-ligation read pairs have strand orientation -+, but not all -+ read pairs were produced by self-ligation.

A distribution estimated from all -+ read pairs would not accurately model the distribution of self-ligation read pairs because self-ligation read pairs are much more likely to align within a short distance than inter-ligation read pairs. This is because the fragment length distribution induced by fragmentation limits the distance between which the ends of self-ligation read pairs may align whereas there is no constraint on the distance between which the ends of inter-ligation read pairs may align. To more accurately estimate the distribution of self-ligation read pairs, we weight the contribution of each -+ read pair by the estimated likelihood that the read pair was produced by self-ligation according to the distance between the aligned locations of the read pair ends.

Let *z*_*i*_ indicate whether -+ read pair *r*_*i*_ was produced by self-ligation or inter-ligation and *d*(*r*_*i*_) be the distance between the aligned locations of the ends of -+ read pair *r*_*i*_. The likelihood that -+ read pair *r*_*i*_ was produced by self-ligation according to *d*(*r*_*i*_) can be expressed in terms of quantities that can be estimated from the data

$$\begin{array}{c} \mathrm{Pr}\left(z_{i}=self|d\left(r_{i}\right)\right)=\frac{\mathrm{Pr}\left(d\left(r_{i}\right)|z_{i}=self\right)\mathrm{Pr}\left(z_{i}=self\right)}{\mathrm{Pr}(d\left(r_{i}\right))} \end{array}$$ (1)

Pr(*d*(*r*_*i*_)) for all -+ read pairs can be estimated by applying an unweighted kernel approach

$$\begin{array}{c} \hat{\mathrm{Pr}}\left(d\left(r\right)=x\right)=\sum_{i=1}^{N_{-+}}\frac{1}{h_{-+}N_{-+}}K_{1}(\frac{x-d(r_{i})}{h_{-+}}) \end{array}$$ (2)

*N*_−+_ is the total number of -+ read pairs and *K*_1_ is a standard univariate Gaussian distribution. The bandwidth *h*_−+_ is a parameter that controls the trade-off between fitting the training data and discovering a smooth estimate. To choose an appropriate *h*_−+_ we use a least-squares cross-validation approach that minimizes the integrated square error (*ISE*) of $\hat{\text{Pr}}(x)$.

$$\begin{array}{c} ISE\left(\hat{f}\right)=\int {\left(\hat{f}-f\right)}^{2} \end{array}$$ (3)

The $ISE(\hat{\text{Pr}}(d(r)=x))$ can be approximately minimized by minimizing for all -+ read pairs [22]

$$\begin{array}{c} \sum_{i}\sum_{j}\frac{1}{\sqrt{2}h_{-+}}K_{1}(\frac{d\left(r_{i}\right)-d\left(r_{j}\right)}{\sqrt{2}h_{-+}})-\frac{2}{N_{-+}}\sum_{i}[\frac{\hat{\mathrm{Pr}}\left(d\left(r_{i}\right)\right)-\frac{1}{\sqrt{2\pi }}}{N_{-+}-1}] \end{array}$$ (4)

We cannot estimate Pr(*d*(*r*_*i*_)|*z*_*i*_ = *self*) directly for the same reason that we cannot estimate the self-ligation read pair distribution directly. We can estimate Pr(*d*(*r*_*i*_)|*z*_*i*_ = *inter*) directly because all non -+ read pairs are produced by inter-ligation. We also apply an unweighted kernel approach to estimate this distribution

$$\begin{array}{c} \hat{\mathrm{Pr}}\left(d\left(r\right)=x|z=inter\right)=\sum_{i=1}^{N_{non-+}}\frac{1}{h_{non-+}N_{non-+}}K_{1}(\frac{x-d(r_{i})}{h_{non-+}}) \end{array}$$ (5)

We choose an appropriate *h*_*non*−+_ by approximately minimizing the $ISE(\hat{\text{Pr}}(d(r)=x|z=inter))$.

Given estimates for Pr(*d*(*r*_*i*_)) and Pr(*d*(*r*_*i*_)|*z*_*i*_ = *inter*), we can estimate Pr(*d*(*r*_*i*_)|*z*_*i*_ = *self*) by assuming that Pr(*d*(*r*_*i*_)) is a mixture of the distributions Pr(*d*(*r*_*i*_)|*z*_*i*_ = *self*) and Pr(*d*(*r*_*i*_)|*z*_*i*_ = *inter*)

$$\begin{array}{ccc} \mathrm{Pr}(d\left(r_{i}\right)) & = & \mathrm{Pr}\left(z_{i}=self\right)\mathrm{Pr}\left(d\left(r_{i}\right)|z_{i}=self\right)\\ & & +\mathrm{Pr}\left(z_{i}=inter\right)\mathrm{Pr}\left(d\left(r_{i}\right)|z_{i}=inter\right) \end{array}$$ (6)

By rearranging the terms in this equation we can obtain

$$\begin{array}{c} \mathrm{Pr}(d\left(r_{i}\right)|z_{i}=self)=\\ \;\frac{\mathrm{Pr}\left(d\left(r_{i}\right)\right)-\mathrm{Pr}\left(z_{i}=inter\right)\mathrm{Pr}\left(d\left(r_{i}\right)|z_{i}=inter\right)}{\mathrm{Pr}(z_{i}=self)} \end{array}$$ (7)

The final missing component is Pr(*z*_*i*_ = *self*) = 1 − Pr(*z*_*i*_ = *inter*). We assume that the average number of read pairs with each of the three strand orientations other than -+ is a good estimator for the number of -+ read pairs that were produced by inter-ligation. We use this information to estimate Pr(*z*_*i*_ = *inter*)

$$\begin{array}{c} \hat{\mathrm{Pr}}\left(z_{i}=inter\right)=\frac{\text{avg}.\#\text{non}-+\text{read pairs}}{\#-+\text{read pairs}} \end{array}$$ (8)

This allows us to estimate the self-ligation read pair distribution using a weighted kernel approach weighted by Pr(*z* = *self*|*d*(*r*_*i*_))

$$\begin{array}{c} \hat{\mathrm{Pr}}\left(r=\left\langle x,y\right\rangle |z=self\right)=\sum_{i=1}^{N_{-+}}\frac{\mathrm{Pr}(z=self|d\left(r_{i}\right))}{h_{self}}K_{2}(\frac{\left\langle x,y\right\rangle -r_{i}}{h_{self}}) \end{array}$$ (9)

where in this case *K*_2_ is a bivariate standard Gaussian distribution with no correlation between the dimensions. To choose an appropriate bandwidth *h*_*self*_ we approximately minimize $ISE(\hat{\text{Pr}}(r=\langle x,y\rangle |z=self))$ by minimizing

$$\begin{array}{c} \sum_{i}\sum_{j}\frac{\mathrm{Pr}\left(z=self|d\left(r_{i}\right)\right)\mathrm{Pr}\left(z=self|d\left(r_{j}\right)\right)}{\sqrt{2}h_{self}}K_{2}(\frac{r_{i}-r_{j}}{\sqrt{2}h_{self}})\\ -\frac{2}{N}\sum_{i}[\frac{\hat{\mathrm{Pr}}\left(r_{i}|z_{i}=self\right)-\frac{\mathrm{Pr}(z=self|d\left(r_{i}\right))}{\sqrt{2\pi }}}{\sum_{j\neq i}\mathrm{Pr}\left(z=self|d\left(r_{j}\right)\right)}] \end{array}$$ (10)

#### Estimating the 1D Marginal Distribution of Protein Occupancy

We assume that the self-ligation read pair distribution is the result of the convolution of the marginal distribution of protein occupancy and a distribution that models DNA fragmentation which we will refer to as the read spread function (*RSF*). If we let *q* be the genomic location occupied by the protein,

$$\begin{array}{c} \mathrm{Pr}\left(r=\left\langle x,y\right\rangle |z=self\right)=\sum_{u}\mathrm{Pr}\left(q=u\right)RSF\left(\left\langle x-u,y-u\right\rangle \right) \end{array}$$ (11)

Simultaneously deconvolving the marginal distribution of protein occupancy and the *RSF* from the self-ligation read pair distribution is an example of a blind deconvolution problem. This problem commonly arises in the context of image processing. It is often the case that a camera will systematically blur the images that it captures because of flaws in its lens. This blurring process is modeled as a convolution of the distribution of light that enters the camera lens with a point spread function (*PSF*) that is induced by the flaws in the lens. The *PSF* specifically describes the effect that the lens flaws will have on a theoretical point source of light. In our case, the RSF describes the manner in which self-ligation read pairs are likely to be distributed given the theoretical occupancy of the protein at a genomic location.

If we assume at first that the *RSF* is known, the marginal distribution of protein occupancy can be approximately recovered using a standard approach known as Richardson-Lucy (RL) deconvolution [23, 24]. The RL algorithm iteratively applies the following EM-like update

$$\begin{array}{ccc} & & {\hat{\mathrm{Pr}}}_{i+1}\left(q=u\right)=\\ & & {\hat{\mathrm{Pr}}}_{i}\left(q=u\right)\{\sum_{x}\sum_{y}[\frac{\hat{\mathrm{Pr}}\left(r=\left\langle x,y\right\rangle |z=self\right)}{\sum_{v}{\hat{\mathrm{Pr}}}_{i}\left(q=v\right)RSF\left(\left\langle x-v,y-v\right\rangle \right)}]RSF\left(-\left\langle x-u,y-u\right\rangle \right)\} \end{array}$$ (12)

RL deconvolution has been shown empirically to converge to a maximum-likelihood estimate for Pr(*q* = *u*) and preserves the non-negativity and sum of the initial guess Pr_0_(*q* = *u*). To extend RL deconvolution to the blind case, we take an approach similar to that proposed in [25] and alternate the updates described by Eq 12 with the following updates

$$\begin{array}{ccc} & & {\hat{RSF}}_{i+1}\left(\left\langle x,y\right\rangle \right)=\\ & & {\hat{RSF}}_{i}\left(\left\langle x,y\right\rangle \right)\{\sum_{u}[\frac{\hat{\mathrm{Pr}}\left(r=\left\langle x-u,y-u\right\rangle |z=self\right)}{\sum_{v}{\hat{RSF}}_{i}\left(\left\langle x-u-v,y-u-v\right\rangle \right)\hat{\mathrm{Pr}}\left(q=v\right)}]\hat{\mathrm{Pr}}\left(q=-u\right)\} \end{array}$$ (13)

**Fig 2:**
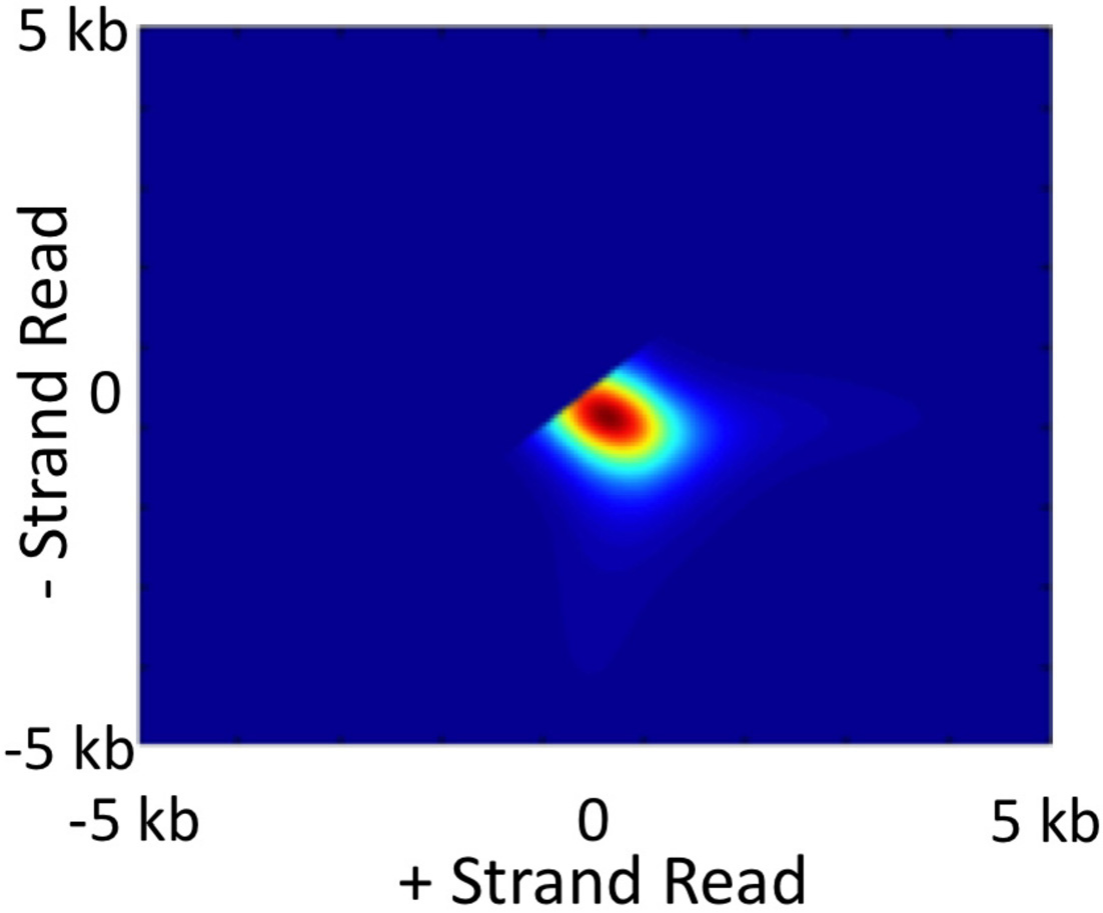
A typical read spread function estimated from RNA PolII ChIA-PET data.

The overall procedure then entails going back and forth between updating $\hat{\text{Pr}}(q=u)$ for several iterations while holding $\hat{RSF}(\langle x-u,y-u\rangle )$ fixed and then updating $\hat{RSF}(\langle x-u,y-u\rangle )$ for several iterations while holding $\hat{\text{Pr}}(q=u)$ fixed. Despite the unconstrained nature of the blind deconvolution approach, the recovered *RSF* conforms to our expectations. The *RSF* in Fig 2 is typical of what is recovered from RNA PolII ChIA-PET data. Given a location bound by the protein, we would expect the most likely alignment of the ends of self-ligation read pairs to be roughly equidistant to the occupied location with the distance from the occupied location determine by the degree of fragmentation. The typical *RSF* that we estimate has the greatest value along the line through the origin that is perpendicular to the identity line. Points along this line reflect self-ligation read pairs that align equidistantly to the occupied location which is represented by the origin in the *RSF*. The distance of the peak in the *RSF* from the origin reflects the most likely fragment size generated by the sonication step. Thus, the *RSF* that we recover using our blind deconvolution approach conforms to our expectations and provides useful information about the fragmentation step of the ChIP procedure.

#### Efficiently estimating the genome-wide protein occupancy distribution

RL blind deconvolution works well for deconvolving the protein occupancy distribution for regions of the genome that are on the order of megabases in size. However, the time that it would take to deconvolve the full genome-wide distribution of protein occupancy is impractical. Based on observations made about typical *RSF*s estimated by RL blind deconvolution from portions of real ChIA-PET datasets, we devised a highly efficient procedure that achieves a level of accuracy comparable to full RL blind deconvolution. The observation we made was that typical *RSF*s estimated by RL blind deconvolution from portions of real datasets are unimodal and sharply peaked. This implies that the *RSF* can be approximated by a function with all of its mass at the peak of the *RSF*. This approximation allows for a very efficient deconvolution procedure. If the peak of the estimated *RSF* is at ⟨−*λ*, *λ*⟩, we estimate the protein occupancy distribution as

$$\begin{array}{c} \hat{\mathrm{Pr}}\left(q=u\right)\propto \hat{\mathrm{Pr}}\left(r=\left\langle u-\lambda ,u+\lambda \right\rangle |z=self\right) \end{array}$$ (14)

In summary, to estimate the marginal distribution of protein occupancy from a full genome-wide ChIA-PET dataset we first estimate the genome-wide self-ligation read pair distribution. We then apply RL blind deconvolution to a 5 megabase region of the genome to obtain a good estimate for the *RSF*. Finally, we identify the peak of the estimated *RSF* and estimate the distribution of RNA PolII occupancy as in (Eq 14).

#### Estimating the 2D Joint Distribution of Protein Occupancy

Chromatin looping allows proteins to simultaneously occupy two genomic locations [26]. Inter-ligation read pairs can be thought of as samples from a joint distribution of protein occupancy with positional noise introduced by fragmentation. We make several assumptions about this process. We assume that the inter-ligation read pairs are based on independent samples from the joint distribution of protein occupancy. We associate the lower coordinate protein location *q* ^(1)^ with the lower coordinate end of the read pair *r* ^(1)^ and the higher coordinate protein location *q* ^(2)^ with the higher coordinate end of the read pair *r* ^(2)^.

$$\begin{array}{ccc} \mathrm{Pr}(q=\left\langle u,v\right\rangle |R_{inter}) & = & \frac{1}{N_{inter}}\sum_{r_{i}\in R_{inter}}\mathrm{Pr}\left(q=\left\langle u,v\right\rangle |\left\langle r_{i}^{\left(1\right)},r_{i}^{\left(2\right)}\right\rangle \right) \end{array}$$ (15)

$$\begin{array}{cc} = & \frac{1}{N_{inter}}\sum_{r_{i}\in R_{inter}}\mathrm{Pr}\left(q^{\left(1\right)}=u|\left\langle r_{i}^{\left(1\right)},r_{i}^{\left(2\right)}\right\rangle \right)\mathrm{Pr}\left(q^{\left(2\right)}=v|q^{\left(1\right)}=u,\left\langle r_{i}^{\left(1\right)},r_{i}^{\left(2\right)}\right\rangle \right) \end{array}$$ (16)

$$\begin{array}{cc} = & \frac{1}{N_{inter}}\sum_{r_{i}\in R_{inter}}\mathrm{Pr}\left(q^{\left(1\right)}=u|r_{i}^{\left(1\right)}\right)\mathrm{Pr}\left(q^{\left(2\right)}=v|q^{\left(1\right)}=u,r_{i}^{\left(2\right)}\right) \end{array}$$ (17)

The last equality reflects an assumption that we make that the location occupied by the protein is independent of the read pair end that it is not associated with. We will demonstrate that these terms are non-zero in only a relatively small window around their associated read pair end and that the non-associated read pair end has minimal effect on the manner in which we compute these terms. We transform the first term within the sum into quantities that we can compute using Bayes’ Theorem

$$\begin{array}{c} \mathrm{Pr}\left(q^{\left(1\right)}=u|r_{i}^{\left(1\right)}\right)=\frac{\mathrm{Pr}\left(r_{i}^{\left(1\right)}|q^{\left(1\right)}=u\right)\mathrm{Pr}\left(q^{\left(1\right)}=u\right)}{\mathrm{Pr}(r_{i}^{\left(1\right)})} \end{array}$$ (18)

We assume that we can obtain $\text{Pr}(r_{i}^{\left(1\right)}|q^{\left(1\right)}=u)$ by marginalizing the *RSF* that was estimated during the blind deconvolution step. For read pair ends that align to the—strand

$$\begin{array}{c} \mathrm{Pr}\left(r_{i}^{\left(\cdot \right)}|q^{\left(\cdot \right)}=u\right)=\sum_{y}RSF\left(\left\langle r_{i}^{\left(\cdot \right)}-u,y-u\right\rangle \right) \end{array}$$ (19)

Correspondingly, for read pair ends that align to the + strand

$$\begin{array}{c} \mathrm{Pr}\left(r_{i}^{\left(\cdot \right)}|q^{\left(\cdot \right)}=u\right)=\sum_{x}RSF\left(x-u,r_{i}^{\left(\cdot \right)}-u\right\rangle ) \end{array}$$ (20)

Pr(*q* ^(1)^ = *u*) is the distribution of protein marginal occupancy that was estimated in the previous step. The prior read distribution $\text{Pr}(r_{i}^{\left(1\right)})$ reflects any factors that might influence the alignment of reads to locations in the genome. Such factors might include the uniqueness of the sequence around that location in the genome and bias in the library preparation or sequencing for the sequence around that location. We assume that $\text{Pr}(r_{i}^{\left(1\right)})$ is uniform in this work. However, future work may be improved by utilizing a more informative prior distribution.

We also transform the second term within the sum in (Eq 17) using Bayes’ Theorem

$$\begin{array}{ccc} \mathrm{Pr}(q^{\left(2\right)}=v|q^{\left(1\right)}=u,r_{i}^{\left(2\right)}) & = & \frac{\mathrm{Pr}\left(r_{i}^{\left(2\right)}|q^{\left(1\right)}=u,q^{\left(2\right)}=v\right)\mathrm{Pr}\left(q^{\left(2\right)}=v|q^{\left(1\right)}=u\right)}{\mathrm{Pr}(r_{i}^{\left(2\right)}|q^{\left(1\right)}=u)} \end{array}$$ (21)

$$\begin{array}{ccc} & & \approx \frac{\mathrm{Pr}\left(r_{i}^{\left(2\right)}|q^{\left(2\right)}=v\right)\mathrm{Pr}\left(q^{\left(2\right)}=v\right)}{\mathrm{Pr}(r_{i}^{\left(2\right)})} \end{array}$$ (22)

The approximation in (Eq 22) incorporates assumptions to simplify all terms involved. We assume that $r_{i}^{\left(2\right)}$ only depends on the location of protein occupancy that it is associated with, and hence $\text{Pr}(r_{i}^{\left(2\right)}|q^{\left(1\right)}=u,q^{\left(2\right)}=v)\approx \text{Pr}(r_{i}^{\left(2\right)}|q^{\left(2\right)}=v)$ which we obtain by marginalizing the estimated *RSF*. We next assume that *q* ^(1)^ and *q* ^(2)^ are independent. This is clearly not true, since otherwise we would have no need of estimating their joint distribution. But, since $\text{Pr}(r_{i}^{\left(2\right)}|q^{\left(2\right)}=v)$ is only non-zero in a relatively small range around *v*, the purpose of Pr(*q* ^(2)^ = *v*|*q* ^(1)^ = *u*) is mainly to fine tune the probability that *q* ^(2)^ = *v* if $r_{i}^{\left(2\right)}$ falls within that range. We expect the locations of peaks of Pr(*q* ^(2)^ = *v*|*q* ^(1)^ = *u*) to roughly agree with peaks of Pr(*q* ^(2)^ = *v*) if they exist, and so we assume that we can swap one for the other in this case. Finally, we assume that $r_{i}^{\left(2\right)}$ is independent of the location of protein occupancy that it is not associated with, allowing us to substitute $\text{Pr}(r_{i}^{\left(2\right)})$ for $\text{Pr}(r_{i}^{\left(2\right)}|q^{\left(1\right)}=u)$.

These transformations allow us to write the estimated joint distribution of protein occupancy as

$$\begin{array}{c} \hat{\mathrm{Pr}}\left(q=\left\langle u,v\right\rangle |R_{inter}\right)\propto \sum_{r_{i}\in R_{inter}}\mathrm{Pr}\left(r_{i}^{\left(1\right)}|q^{\left(1\right)}=u\right)\mathrm{Pr}\left(q^{\left(1\right)}=u\right)\mathrm{Pr}\left(r_{i}^{\left(2\right)}|q^{\left(2\right)}=v\right)\mathrm{Pr}\left(q^{\left(2\right)}=v\right) \end{array}$$ (23)

#### *Germ* ^*X*^: Estimating the Conditional Distribution of Protein Occupancy with a Set of Locations *X*

In many situations we are interested in estimating the joint occupancy of a protein with a set of genomic locations *X*. For example, when analyzing RNA PolII ChIA-PET data, a common query might be to detect regions that are jointly occupied by RNA PolII along with a location from set of annotated transcription start sites (TSSs). If we define *TSS* to be a set of annotated TSSs, we refer to *Germ* ^*TSS*^ as the process of estimating Pr(*q* = ⟨*u*, *v*⟩|*R*_*inter*_) only for *v* ∈ *TSS*.

#### Evaluating the Significance of Portions of Estimated Distributions of Marginal and Joint Protein Occupancy

Once we have estimated distributions of marginal and joint protein occupancy from ChIA-PET data we evaluate the significance of the estimated protein occupancy within a given region or the joint occupancy within a given pair of regions. We describe our approach as applied to a marginal distribution of protein occupancy and then extend the approach to joint distributions. Given a genomic region *reg* of size *w* base pairs, let $p=\sum_{u\in reg}\hat{\text{Pr}}(q=u)$. If we let *Z* ∼ Binomial(*N*_*self*_, *p*) and $Y∼\text{Binomial}(N_{self},\frac{w}{M})$ where *M* is the size of the mappable genome, we then evaluate the significance of the protein occupancy within *reg* as Pr(*Y* > *Z*). In other words, we calculate the probability that more self-ligation read pairs would be associated with *reg* according to a uniform distribution of protein occupancy than would be associated with *reg* according to the estimated distribution of protein occupancy.

We extend this approach to evaluating the significance of pairs of regions according to a joint distribution of protein occupancy. Given a pair of regions *reg*_*a*_ and *reg*_*b*_, let $p_{joint}=\sum_{u\in reg_{a}}\sum_{v\in reg_{b}}\hat{\text{Pr}}(q=\langle u,v\rangle |R_{inter})$, $p_{a}=\sum_{u\in reg_{a}}\hat{\text{Pr}}(q=u)$, and $p_{b}=\sum_{u\in reg_{b}}\hat{\text{Pr}}(q=u)$. If we then let *Z* ∼ Binomial(*N*_*inter*_, *p*_*joint*_) and *Y* ∼ Binomial(*N*_*inter*_, *p*_*a*_ *p*_*b*_), we then evaluate the significance of the joint protein occupancy of the regions *reg*_*a*_ and *reg*_*b*_ as Pr(*Y* > *Z*).

#### Significance evaluation for *Germ* ^*X*^

The estimate $\hat{\text{Pr}}(q=\langle u,v\rangle |R_{inter})$ for *v* ∈ *X* that is obtained by applying *Germ* ^*X*^ is void of mass for much of its domain. This is because not enough inter-ligation read pairs can be sequenced to fully explore this space given current technologies. Without considering the mass that is missing from the estimate of $\hat{\text{Pr}}(q=\langle u,v\rangle |R_{inter})$, the significance of portions of the distribution for which mass is estimated will be overestimated. To remedy this issue, we introduce a method for estimating how much mass is missing from the estimate of $\hat{\text{Pr}}(q=\langle u,v\rangle |R_{inter})$ in order to more accurately evaluate the significance of portions of this distribution. We assume an ordering on the *v*_*i*_ ∈ *X* and let $t_{i}=\sum_{u}\hat{\text{Pr}}(q=\langle u,v_{i}\rangle |R_{inter})$ and $m_{i}=\hat{\text{Pr}}(q=v_{i})$. If we assume that there is some amount of mass *τ*_*i*_ that is missing from *t*_*i*_, then we can find a setting of the *τ*_*i*_ such that $\frac{t_{i}+\tau_{i}}{\sum_{i}t_{i}+\tau_{i}}=\frac{m_{i}}{\sum_{i}m_{i}}$. However, there are many valid settings of the *τ*_*i*_ and larger values of the *τ*_*i*_ will cause portions of the estimated distribution to be evaluated as less significant.

To choose an appropriate setting of the *τ*_*i*_ we introduce a procedure that allows us to choose *τ*_*i*_ large enough to avoid overestimating the significance of portions of the estimated distribution. We first choose a set of candidate regions for each *v*_*i*_ ∈ *X* which we will evaluate for significance based on $\hat{\text{Pr}}(q=\langle u,v\rangle |R_{inter})$. We do this by setting a threshold *f* and adding a region *reg* to the set for *v*_*i*_ if $\forall u\in reg,\hat{\text{Pr}}(\langle u,v_{i}\rangle |R_{inter})>f$. We then identify an *i*_*max*_ such that ∀*i*, *t*_*i*_*max*__ ≥ *t*_*i*_. We choose some *c* > 1 and set *τ*_*i*_*max*__ = (*c* − 1)*t*_*i*_*max*__. We hold *τ*_*i*_*max*__ fixed and apply an iterative procedure to find settings for *τ*_*i*_ (*i* ≠ *i*_*max*_) such that $\frac{t_{i}+\tau_{i}}{\sum_{i}t_{i}+\tau_{i}}=\frac{m_{i}}{\sum_{i}m_{i}}$. For each iteration, we cycle through *i* ≠ *i*_*max*_ and compute

$$\begin{array}{c} \tau_{i}=\frac{m_{i}\sum_{j\neq i}(t_{j}+\tau_{j})}{\sum_{j\neq i}m_{j}} \end{array}$$ (24)

Once this converges, we evaluate the significance of the regions defined using the threshold *f* in the following way. For a region *reg* in the set for *v*_*i*_ we let $p=\frac{\sum_{u\in reg}\hat{\text{Pr}}(\langle u,v_{i}\rangle |R_{inter})}{t_{i}+\tau_{i}}$ and $p\prime =\sum_{u\in reg}\hat{\text{Pr}}(u)$. If we then let *Z* ∼ Binomial(*N*_*inter*_, *p*) and *Y* ∼ Binomial(*N*_*inter*_, *p*′), the significance of the estimated joint protein occupancy of *v*_*i*_ and *reg* is Pr(*Y* > *Z*). We evaluate the significance of the regions in the sets for all *v* ∈ *X* and identify the regions that have an associated Pr(*Y* > *Z*) less than some threshold such as 0.05. We call these regions significant. For each region, we also note the number of read pairs in *R*_*inter*_ that contributed to *p* for that region. If the ratio of the number of significant regions supported by only one read pair to the total number of significant regions is greater than some target threshold, such as 0.1, we increase *c* and begin the process of finding a new set of *τ*_*i*_. If there are too few significant regions supported by one read pair with Pr(*Y* > *Z*) < 0.05 we reduce *c* and find new *τ*_*i*_. In this manner we search for *c* that achieves a target fraction of weakly supported jointly occupied regions within the set of all regions that evaluate as significant.

## Evaluation

### *Germ* identifies locations involved in interactions at high spatial resolution

**Fig 3:**
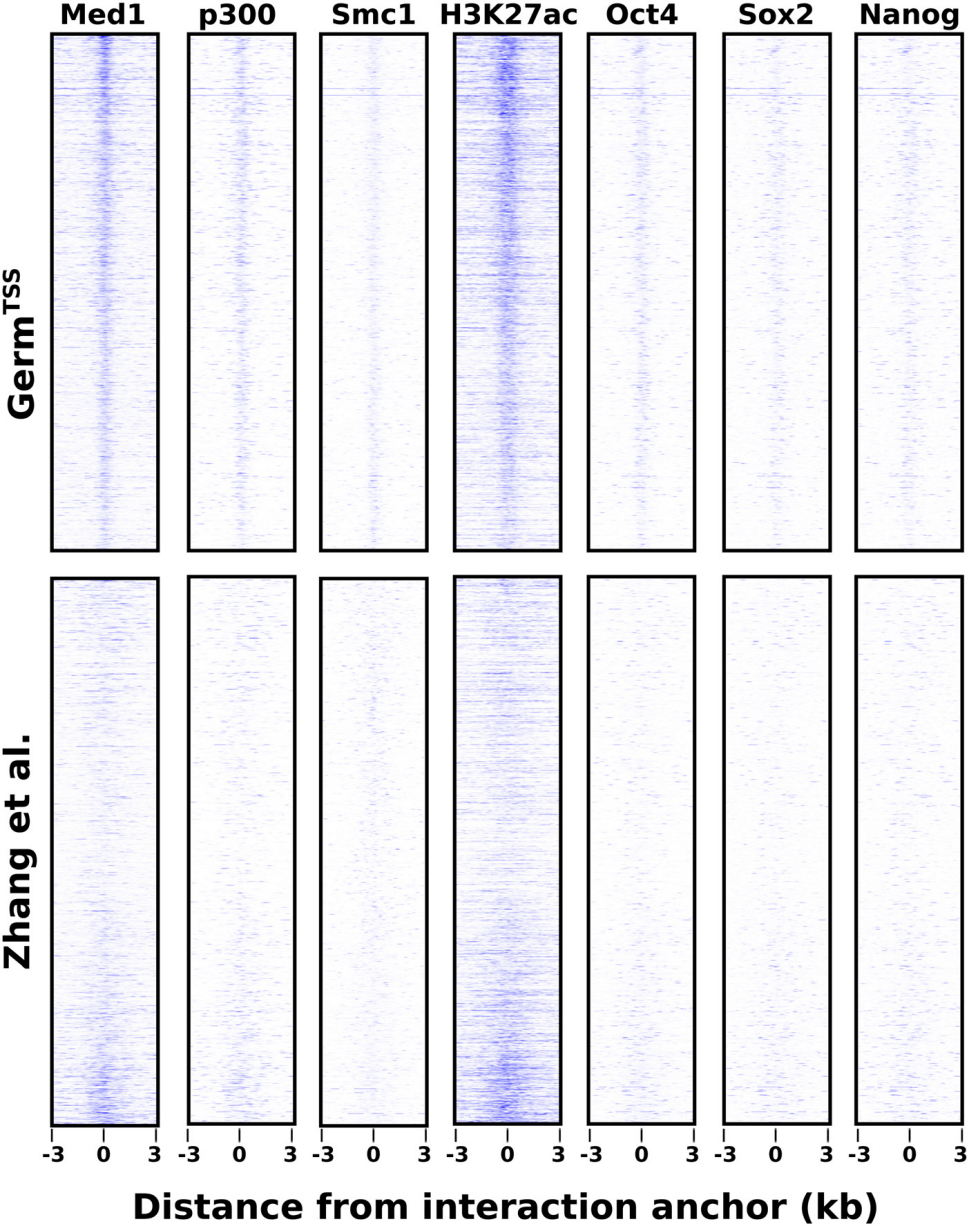
Visualization of ChIP-Seq data in regions detected to interact with TSSs. The top row of boxes contains *TSS*-distal, *TSS* jointly occupied regions identified by *Germ* ^*TSS*^. The bottom row of boxes contains the corresponding regions from [27]. The 6 kilobase regions are centered on the estimated *eloc* or midpoint and are ordered by the significance associated with the interaction. Each column represents data from a ChIP-Seq dataset that is associated with active enhancers.

We applied *Germ* to PolII ChIA-PET data from ESCs [27] to identify locations that interact with TSSs. By examining ChIP-Seq data for several features of active enhancers at the locations that *Germ* detects as interacting with TSSs we found that these locations align closely with locations that appear to be active enhancers. We incorporated a set of annotated TSSs from the UCSC knownGene database to profile the occupancy of PolII conditioned on the locations of the annotated TSSs. For each TSS, *Germ* provided a set of regions that are jointly occupied by PolII along with the TSS. The joint occupation of a region with a TSS by PolII indicates that this region is spatially proximal to the TSS and that PolII is also present at the junction between the region and the TSS. PolII tends to occupy relatively broad regions of the genome, but upon examining the distributions of PolII occupancy that we estimate with *Germ*, we observed that regions of elevated occupancy generally contain locations with locally maximal likelihood of occupancy. We noted the location within each TSS-interacting region that *Germ* determines to be the most likely anchor point for the interaction. As shown in Fig 3, the *Germ* estimated anchor points are informative in that they align closely with maximal locations of enrichment for active enhancer-related ChIP-Seq data.

**Fig 4:**
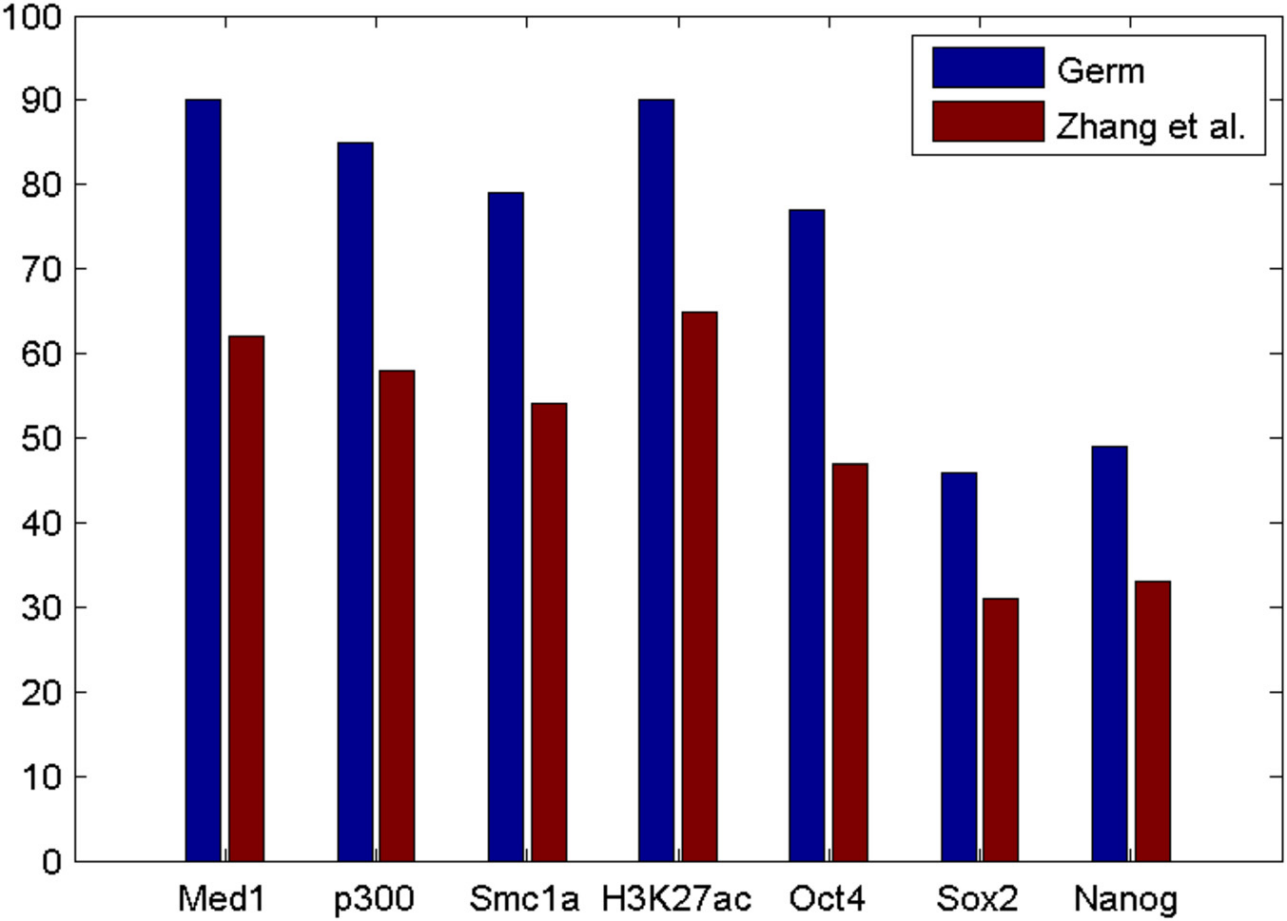
The percentages of the locations identified by *Germ* and the ChIA-PET Tool that are enriched for each of the ChIP-Seq datasets.

The difficulty in extracting locations that interact with TSSs from results obtained using the ChIA-PET Tool highlights the superior informativeness of *Germ* results. We obtained the set of interactions called by the ChIA-PET Tool from the same ChIA-PET data and filtered out the interactions that do not contain a TSS within either anchor region. Since the ChIA-PET Tool interactions do not include estimates of the most likely locations within the anchor regions that are jointly occupied by RNA PolII, we chose the midpoint of each anchor region as the approximate maximally occupied location. We further filtered the interactions to identify the set of interactions that contain a TSS within one anchor region and for which the midpoint of the other anchor region is at least 2kb away from any TSS. As shown in Fig 3, the locations identified in this way are not as closely associated with the ChIP-Seq data as the locations identified with *Germ*. To quantify the enhancer properties at the locations identified by *Germ* and the ChIA-PET Tool we identified 500 bp windows centered on the locations identified by the two methods. We examined the significance of enrichment for each of the ChIP-Seq data within each of the identified windows as shown in Fig 4. The two methods identified similar numbers of TSS-interacting locations (*Germ* identified 2924 and the ChIA-PET Tool identified 3098). The greater percentage of significantly enriched locations within the *Germ* identified locations for all of the ChIP-Seq data emphasizes the usefulness of analyzing PolII ChIA-PET data with *Germ* for the purpose of identifying active enhancers.

## Results

### *Germ* discovers meaningful interactions involving TSSs

**Fig 5:**
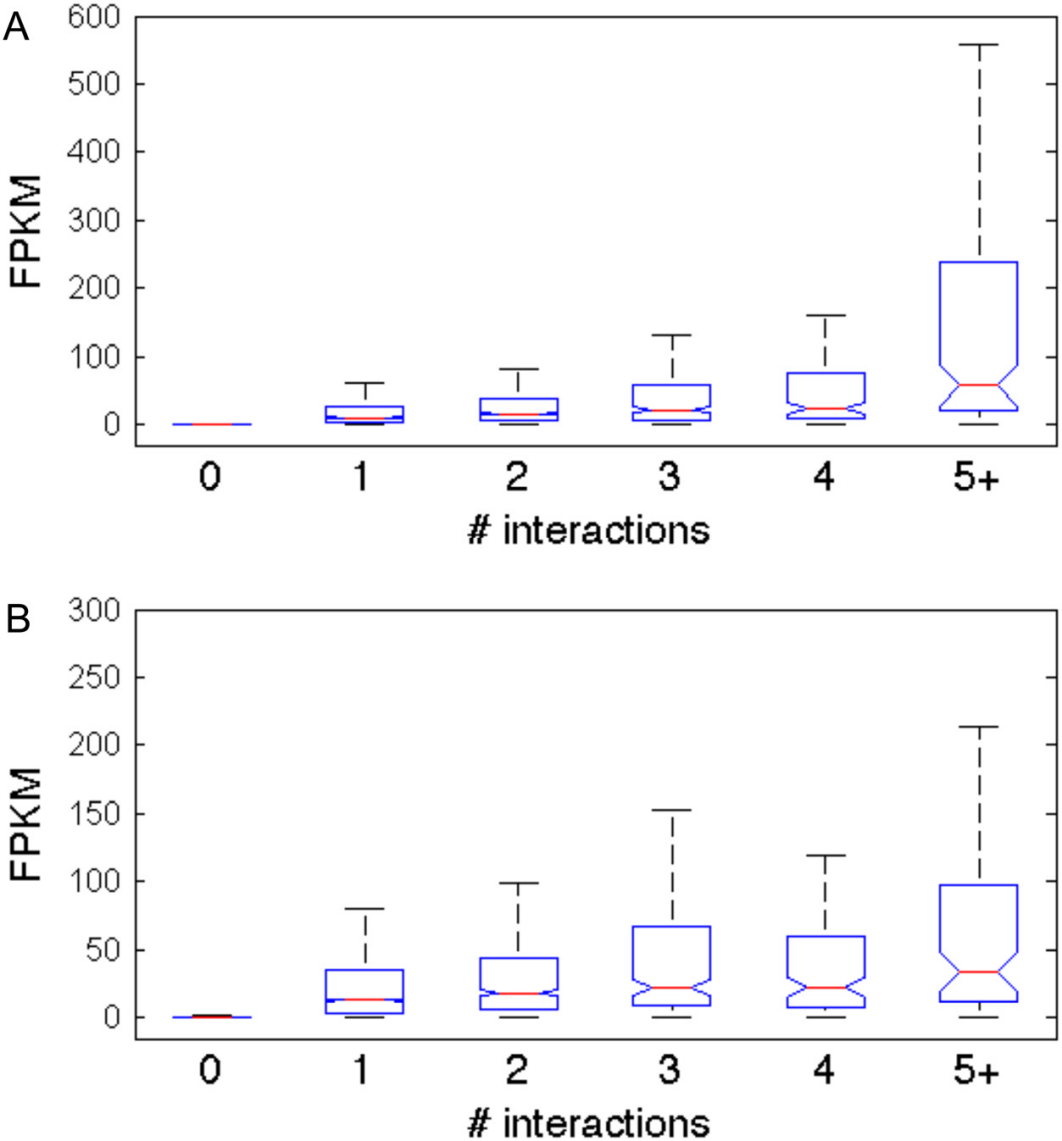
Transcription levels are correlated with the number of nonTSS locations with which a TSS interacts. Genes are categorized based on the number of nonTSS locations that their TSSs interact with in (A) ESCs and (B) pMNs. The boxplots reflect the distribution of FPKM values computed for the genes in each group from RNA-Seq data.

Since *Germ* identifies TSS-interacting locations that align closely with enhancer related ChIP-Seq data, we decided to investigate whether the interactions detected by *Germ* appear to influence the expression levels of the genes involved. We performed PolII ChIA-PET with motor neuron progenitors (pMNs) and applied *Germ* in order to characterize enhancers that are differentially utilized between pMNs and ESCs. We also performed RNA-Seq to profile transcription levels of genes in both cell types. We hypothesized that the interactions that *Germ* identifies between TSSs and locations that are more than 2 kb away from any TSS reflect functional interactions between enhancers and promoters. We call such interactions TSS-nonTSS interactions. As shown in Fig 5, genes involved in TSS-nonTSS interactions exhibit greater levels of transcription than genes not involved in such interactions. The level of transcription is also correlated with the number of TSS-nonTSS interactions that the gene is involved in implying that such interactions may have an additive effect.

**Fig 6:**
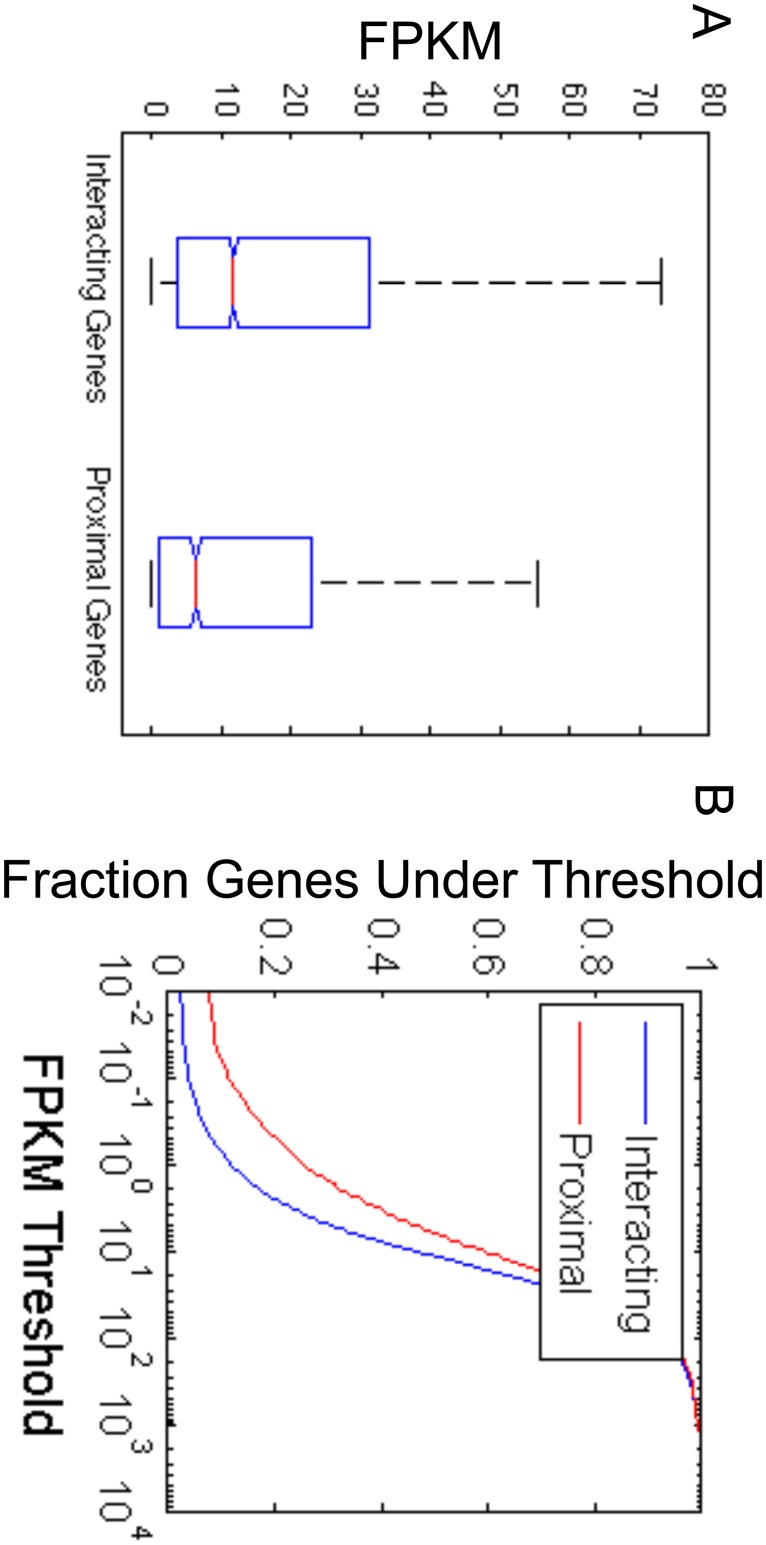
Considering interactions allows more highly transcribed genes to be identified than the set of genes that are closest to the locations that are detected to interact with TSSs. (A) The set of Interacting Genes is the set of genes for which their TSS is identified by *Germ* as interacting with at least one nonTSS location. The set of Proximal Genes is the set of genes for which their TSS is the closest TSS to the set of nonTSS locations that are identified by *Germ* as interacting with at least one TSS. The boxplots reflect the distribution of FPKM values computed for the genes in each group from the ESC RNA-Seq data. (B) The cumulative distributions of the transcription levels of the two sets of genes in ESCs demonstrate that a greater fraction of the genes proximal to the *Germ* identified nonTSS locations have transcription levels less than any FPKM threshold than the set of genes that interact with the nonTSS locations.

The observed correlation between TSS-nonTSS interactions and transcription levels led us to ask whether the existence of nearby active enhancers is enough to induce a TSS-nonTSS interaction and increase transcription levels or if active enhancers specifically target genes that are not necessarily the closest gene. We compared the transcription levels of the genes closest to the locations that *Germ* identifies as involved in TSS-nonTSS interactions to the levels of the genes that are involved in TSS-nonTSS interactions in ESCs. As shown in Fig 6, the genes that are involved in TSS-nonTSS interactions exhibit greater levels of transcription. This indicates that enhancers have specific targets and do not necessarily have the effect of increasing the transcription levels of the genes closest to them.

**Fig 7:**
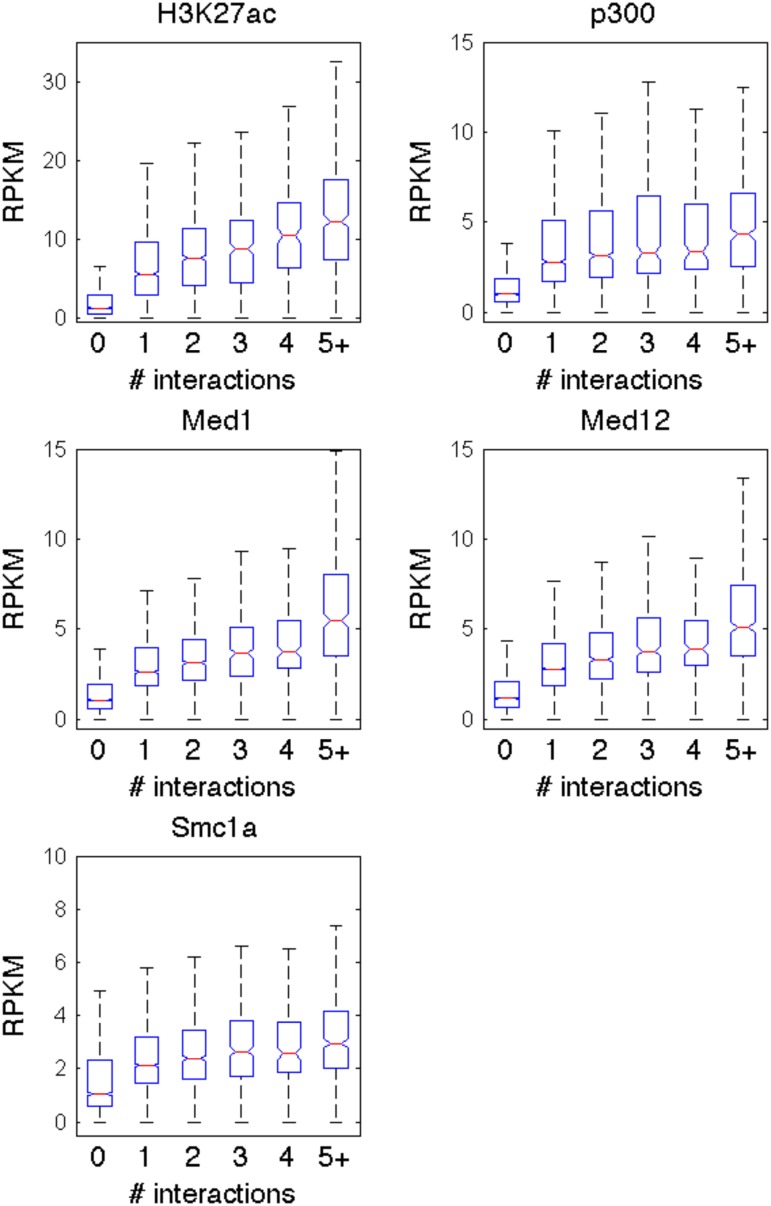
Enrichment for enhancer associated marks is correlated with the number of TSSs with which a nonTSS location interacts. All nonTSS locations that are involved in an interaction with a TSS in at least one of the cell types were considered. The nonTSS locations were categorized based on the number of TSSs that they interact with in ESCs. RPKM values were computed from ChIP-Seq data in 1 kb windows centered on each nonTSS location. The boxplots reflect the distributions of RPKM values for the nonTSS locations in each group for each ChIP-Seq dataset.

We observed that TSS-interacting locations that *Germ* identifies interact with anywhere from one to a hundred or more distinct TSSs. We wondered whether enhancers that target more genes exhibit stronger enhancer characteristics. We collected the locations that interact with TSSs according to *Germ* in either ESCs or pMNs. We grouped these locations based on the number of TSS-nonTSS interactions in which they are involved in ESCs. As shown in Fig 7, the degrees of enrichment for H3K27ac, Med1, Med12, p300, and Smc1a all correlate with the number of interactions in which a location is involved. This suggests that the strength of the active enhancer characteristics at a given location reflects the number of genes targeted by that location.

### Differentially utilized enhancers contain cell type appropriate transcription factor motifs

**Fig 8:**
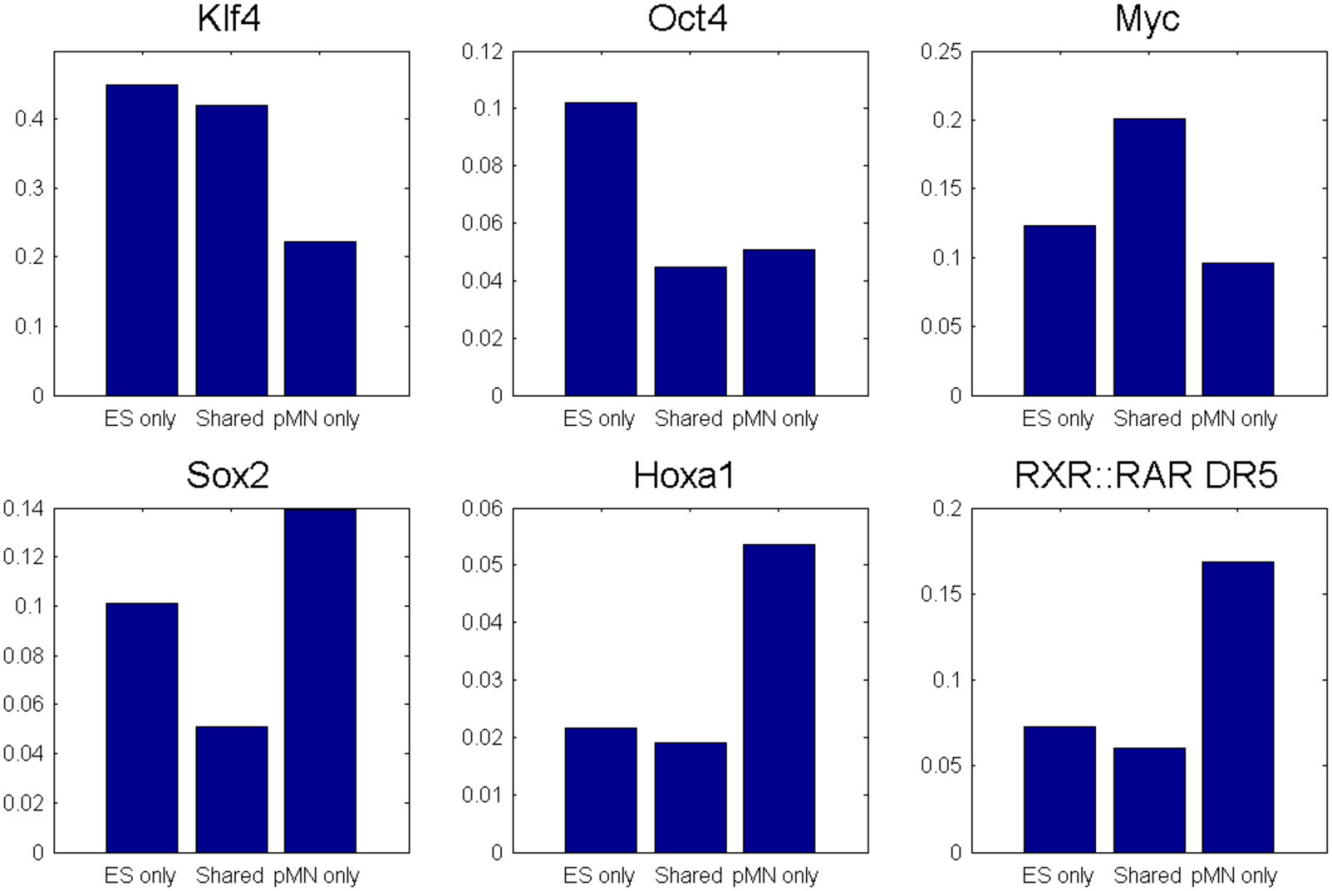
Enhancer usage reflects cell-type appropriate motif enrichment. 1 kb windows centered on Med1 binding events involved in interactions with TSSs in one or both cell types were scanned for matches to known transcription factor motifs. Med1 binding events were categorized based on whether they interact with TSSs in one or both cell types. The bar graphs reflect the percentages of Med1 binding events in each group that have a motif match within 500 bp for several important transcription factors.

Given the evidence that we collected that indicate that the locations that *Germ* identifies as TSS-interacting are active enhancers, we decided to investigate whether the sequence context of *Germ* identified enhancers reflects their cell type specificity. We grouped the *Germ* identified enhancers according to their cell type utilization resulting in 2,217 enhancers that are only utilized in ESCs, 950 that are only utilized in pMNs, and 314 that are utilized in both cell types. We tested for the presence of several sequence motifs corresponding to the binding preferences of several transcription factors that are relevant to one or both cell types in 1 kb windows centered on the enhancer locations. We observed interesting patterns of motif presence for many of the factors as shown in Fig 8. The stem cell factor Klf4 [28] motif is present in almost half of the ESC enhancers, and is the most common motif present in these enhancers. Both the Klf4 and Oct4 [29] motifs are present in about twice the percentage of ESC specific enhancers as they are in pMN specific and shared enhancers. pMN specific enhancers are enriched for the RXR::RAR [30] motif and many of the Hox [31] factor motifs compared to ESC specific enhancers. Interestingly, the Sox2 [32, 33] motif is at least twice as common in enhancers specific to either cell type as in the shared enhancers. Sox2 is an important transcription factor for both cell types and it may be the case that the two cell types utilize mostly non-overlapping sets of Sox2 binding events to regulate gene expression.

## Conclusion

We have demonstrated that applying the *Germ* algorithm to ChIA-PET data successfully recovers genomic regions that are enriched for enhancer-related ChIP-Seq data. Their identity as enhancers is further supported by the observation that the interactions that we identify between these regions and TSSs are correlated with transcription levels. Technologies for profiling chromatin interactions genome-wide such as ChIA-PET, Hi-C, and 5C have yet to reach maturity and present analytical challenges such as inherently high false negative rates. Our observations suggest that gene regulation by long-range chromatin interactions with enhancers is a highly dynamic process. Genes that are expressed in more than one cell type may utilize different enhancers to maintain or adjust their expression. This hypothesis is supported by the observation that differentially utilized enhancers contain varying sets of motifs that are recognized by cell-type appropriate transcription factors. The observation that the relationships between enhancers and genes may be not fixed between cell types has been previously noted [18], although caveats about the high false negative rate inherent to ChIA-PET data have been largely ignored. Theories have been proposed [34–37] which have begun to characterize the principles underlying regulatory relationships in the genome, yet the logic behind the placement of enhancers relative to the genes that they regulate has yet to be fully elucidated. We hope that the observations about enhancer usage that we have characterized in this study will help guide future studies that address these important questions regarding transcriptional regulation.

## Supplementary Methods

### Cell Culture

Hb9::GFP transgenic mouse-derived (HBG3) ESCs were cultured over a layer of neomycin resistant Mitomycin-C-treated fibroblasts (Millipore) in EmbryoMax D-MEM (Millipore) supplemented with 15% ESC-grade fetal bovine serum (Thermo Fisher), l-glutamine (Gibco), 0.1 mM *β*-mercaptoethanol and 100 U ml^-1^ leukemia inhibitory factor. Motor neuron differentiation of ESCs was performed as previously described [38]. Briefly, ESCs were trypsinized (Invitrogen) and seeded at 5 × 105 cells per ml in ANDFK medium (Advanced DMEM/F12:Neurobasal (1:1) Medium, 10% Knockout- SR (vol/vol), Pen/Strep, 2 mM l-glutamine, and 0.1 mM 2-mercaptoethanol) to initiate formation of embryoid bodies (day 0). Medium was exchanged on day 2. Patterning of embryoid bodies was induced by supplementing media on day 2 with 1 *μ*M all-trans retinoic acid (Sigma) and 0.5 *μ*M Smo agonist of hedgehog signaling (SAG, Calbiochem).

### ChIP-Seq

ESC ChIP-Seq sequence data were obtained for H3K27ac, Med1, Med12, Smc1a, and p300 [4, 8]. Sequence reads were aligned to the mouse genome (version mm10) using Bowtie [39]. Only uniquely mapping reads were analyzed further. The GEM algorithm [40] was applied to discover binding events. Reads per kilobase per million reads (RPKM) values were computed by identifying the number of reads that fall within a particular region and dividing by the width of the region in kilobases and by the number of millions of reads in the dataset. Enrichment is computed as the proportion of reads from a dataset that fall within the region. If we let *w* represent the width of the region, *M* represent the size of the mappable genome, *p* be the enrichment in the region, *N* be the number of uniquely mapped reads in the dataset, *Z* ∼ Binomial(*N*, *p*), and $Y∼\text{Binomial}(N,\frac{w}{M})$, then the p-value that we associate with the enrichment in the region is Pr(*Y* > *Z*).

### RNA-Seq

Total RNA from mouse embryonic stem cells or motor neuron progenitors was isolated using Trizol Reagent (Invitrogen). mRNA was isolated and strand specific RNA-Seq was performed following the Illumina Truseq protocol. Read pairs were aligned to the mouse genome (version mm10) using STAR [41]. Fragments per kilobase per million reads (FPKM) values were computed using Cufflinks [42].

### ChIA-PET

ChIA-PET experiments were performed as previously described. Briefly, on the appropriate day of differentiation, embryoid bodies were dissociated in trypsin into single cell suspension. Cells were cross-linked using 1% formaldehyde. Cross-linked chromatin was fragmented by sonication to a size of approximately 300bp. Chromatin complexes were immunoprecipitated with monoclonal anti-RNAPII (Covance, 8WG16) coated protein G Dynabeads (Life Technologies). A small portion of ChIP enriched DNA was eluted from beads for quantification. To prepare ChIA-PET libraries DNA was end polished with T4 DNA polymerase (NEB). To assess the degree of intermolecular proximity ligation end polished DNA was divided into 2 aliquots and each ligated to linkers (A or B). The two samples were then joined together for proximity ligation under dilute conditions. Following ligation samples were treated with Mme1 to release paired end tag (PET) constructs. PET constructs were amplified and submitted to sequencing on Illumina Genome Analyzer II.

### Software availability

Complete Java source code is available from https://github.com/christopherreeder/germ.
